# Label Accuracy and Quality of Select Weight-Loss Dietary Supplements Sold on or near US Military Bases

**DOI:** 10.3390/nu16244369

**Published:** 2024-12-18

**Authors:** Cindy Crawford, Andrea T. Lindsey, Bharathi Avula, Kumar Katragunta, Ikhlas A. Khan, Patricia A. Deuster

**Affiliations:** 1 Consortium for Health and Military Performance, Department of Military and Emergency Medicine, F. Edward Hébert School of Medicine, Uniformed Services University, Bethesda, MD 20814, USA; cindy.crawford.ctr@usuhs.edu (C.C.); patricia.deuster@usuhs.edu (P.A.D.); 2 Henry M. Jackson Foundation for the Advancement of Military Medicine, Inc., Bethesda, MD 20817, USA; 3 National Center for Natural Products Research, School of Pharmacy, University of Mississippi, University, MS 38677, USA; bavula@olemiss.edu (B.A.); kkatragu@olemiss.edu (K.K.); ikhan@olemiss.edu (I.A.K.)

**Keywords:** consumer, military personnel, obesity, overweight, performance, readiness, safety, third-party testing

## Abstract

**Background/Objectives**: Sixty-eight percent of service members are living with overweight or obesity, some who may not consult a healthcare provider when they decide to lose weight. Instead, they often turn to weight-loss dietary supplements for self-care solutions. The purpose of this case series study was to examine the label accuracy and quality of select weight-loss dietary supplements sold on or near US military bases across the country. **Methods**: Weight-loss dietary supplements (*n* = 44) were selected and purchased in GNCs, Exchanges, and Shoppettes across 12 military installations from March 2023 to January 2024. Liquid chromatography-mass spectrometry was used to verify the label accuracy according to the Supplement Facts listed ingredients and whether they contained any ingredients prohibited for use in the military. Product label claims were analyzed using the Operation Supplement Safety (OPSS) Risk Assessment Scorecard. **Results**: Thirty-six products (82%) had inaccurate labels. Twenty-seven (61%) had ingredients listed on the label not detected through analysis. Sixteen products (36%) contained hidden ingredients. The four products purchased within one mile from the base listed multiple prohibited ingredients on the labels, with all detected. Forty (91%) products scored a “no-go” according to the OPSS Scorecard and none contained a third-party certification seal on the label. Multiple stimulants were included in products such that the product safety was unknown. **Conclusions**: The majority of weight-loss dietary supplements analyzed in this case series study had inaccurate labels and were considered a “no-go” according to the Scorecard. Service members should only have access to safe, high-quality dietary supplement products. OPSS is collaborating with the Department of Defense stakeholders to determine the most effective ways for service members to have access to third-party certified products on all military establishments.

## 1. Introduction

Overweight and obesity are major public health issues worldwide and potential threats to overall national security. According to the World Health Organization (2022), one in eight people across the world lives with obesity [1]. In the US, approximately one in three young adults aged 17–24 years is “too heavy to serve in the military,” and 19% of active duty service members were obese as of 2020 [2]. The latest statistic shows that 68% of active duty service members are living with overweight or obesity, which makes this condition a leading disqualifier for military service and a “primary contributor to in-service injuries and medical discharges” [3]. The Department of Defense (DoD) spends approximately USD 1.5 billion annually on healthcare costs related to obesity in addition to the consequential costs of lost duty days for service members due to overweight and obesity [2].

Treatment guidelines for individuals with obesity generally include lifestyle changes related to diet and exercise, in addition to behavioral therapy with a healthcare provider [4]. Some weight-loss prescription drugs approved by the US Food and Drug Administration (FDA) may be considered as part of a treatment plan. Service members must follow service-specific policies when considering any prescription medications: lifestyle intervention is typically required for six months before starting any therapy allowable in the military [5]. However, service members struggling with weight may not engage a healthcare provider due to the social stigma associated with overweight or obesity—a perception of being judged, the fear of losing a promotion, or worse, being separated from service [3]. Instead, service members may believe it is their responsibility to manage their weight and thus turn to weight-loss dietary supplements for self-care solutions [6,7]. In fact, recent studies have shown body weight and composition to be a motivating factor for dietary supplement use in the military [8]. Bukhari et al. found that among US Army personnel using dietary supplements, 43% reported they were trying to lose weight, and 83% scored above 240 points in the Army Physical Fitness Test [9]. Knapik et al.’s study showed that as the body mass index (BMI) increases, so does the number of dietary supplements being consumed and specifically the use of combination-type products advertised for weight loss [10]. Unfortunately, misinformation exists surrounding weight-loss dietary supplements that can lead service members to believe that such products are associated with a low risk from either a positive drug test or adverse event. As such, they do not require any shared decision making with a healthcare provider to ensure safe and informed use.

Weight-loss supplements come with promises of quick and convenient solutions, despite the lack of scientific evidence to support such claims [11,12]. In addition, some products have been found to be mislabeled or contaminated with hidden substances not allowed in dietary supplements [13,14,15]. The FDA has put out public notifications about “tainted weight loss products” to alert consumers that certain products promoted for weight loss contain hidden drugs and other substances that may pose significant health risks, such as dimethylhexylamine (DMHA), 1,3-dimethylamylamine (DMAA), and sibutramine [13]. Adverse events associated with the use of weight-loss supplements have included cardiovascular events, liver injury, and stroke [16,17,18,19,20,21,22,23,24,25,26,27,28,29]. In addition to these health risks, service members are faced with career consequences if their use of dietary supplements results in a positive drug test. A positive drug test may result in separation from service or the loss of a career, in addition to avoidable administrative and investigative costs. These ultimately pose a threat to military readiness, yet the sale of and use of these risky supplements continues in the marketplace [30,31].

Operation Supplement Safety (OPSS) is the US DoD program of record for dietary supplements, health, wellness, and performance products [32]. OPSS provides the education, tools, and resources to help users make informed decisions about such products and help them optimize their health, performance, and careers. While certain rules and regulations related to dietary supplements vary widely across the world, no legal framework or protections are in place to seize mislabeled or adulterated supplements before they are sold. It is essential to understand and monitor the current landscape and the quality of dietary supplements promoted to our service members, so that targeted, appropriate education and resources can be available to help keep them safe from potential harm. Product testing and analysis, through methods of Liquid Chromatography-Quadrupole Time-of-Flight Mass Spectrometry (LC-QToF-MS), help to identify risks, raise awareness, and mitigate problematic dietary supplements that could impact service members’ health and careers.

Most recently, the authors analyzed 30 select supplements marketed for weight loss from online companies offering military discounts [14]. The supplements were selected by searching Google using “weight loss military discount” where the results led specifically to a company website offering military discounts for supplements. The marketing included advertisements accompanied by language such as, “We offer discounts as a way to say thanks” and “to thank you for your service”. Here, 25 of the 30 products had inaccurate labels, 24 were misbranded, 7 had hidden substances, and 10 contained ingredients prohibited for military use. In this case series study, the authors examined the label accuracy and quality of select weight-loss dietary supplements being sold on or near US military bases across the country.

## 2. Materials and Methods

Weight-loss dietary supplements (*n* = 44) were selected and purchased in GNCs, Exchanges, and Shoppettes across 12 military bases from March 2023 to January 2024. Twenty-six were from five different Army bases (including four within one mile from the base), 13 from three Air Force bases, three from two Navy bases, and one each from a Marine Corps and Space Force base. Products were sent to the University of Mississippi’s National Center for Natural Products Research for analysis by liquid chromatography-mass spectrometry. The analysis methods are detailed below in Section 2.1, Section 2.2 and Section 2.3.

The content of each product was compared to the Supplement Facts listed ingredients to determine product label accuracy, whether ingredients were listed on the label but not detected through analysis (misbranded), or ingredients were detected through analysis that were not listed on the label (hidden). Next, the authors checked whether products contained any substances on the DoD Prohibited Dietary Supplement Ingredients List [33] or the World Anti-Doping Agency (WADA) Prohibited List [34]. Not all of WADA S6, stimulants prohibited in competition, are prohibited per DoD Instruction 6130.06, Use of Dietary Supplements in the DoD [35]; therefore, those WADA S6 stimulants were reported separately.

Further, the authors analyzed product label claims by using the OPSS Risk Assessment Scorecard [36,37]. The Scorecard is an educational tool that consists of seven yes/no questions a consumer can answer when reading a label to help screen a supplement for safety. A service member should first examine the Supplement Facts label to check whether a product contains any substance on the DoD Prohibited Dietary Supplement Ingredients List. If there is no prohibited ingredient, a service member can screen the label by looking for whether a BSCG Certified Drug-Free, Informed Sport, NSF Certified Sport, or USP-verified third-party certification seal is present on the product label; whether there are fewer than six ingredients on the Supplement Facts label; whether the label is free of the words proprietary, blend, matrix, or complex; if the consumer can easily pronounce the name of each ingredient on the Supplement Facts label; whether the amount of caffeine listed on the label is 200 mg or less per serving; whether the label is free of questionable claims or statements; and whether all the percent Daily Values (% DV) on the Supplement Facts label are less than 200%. A score of four or more yes answers is “okay”. Less than four is a “no-go”. For research analysis purposes, if a prohibited ingredient was detected on a label, it received an automatic “no-go”.

Finally, the authors compared the current analysis results of weight-loss supplements sold on or near military bases to those sold through online companies specifically offering military discounts as a slight snapshot of the weight-loss dietary supplement landscape to which service members are exposed.

### 2.1. Chemicals and Reagents

HPLC-grade acetonitrile, methanol, and formic acid were purchased from Fisher Scientific (Fair Lawn, NJ, USA). Water was obtained using a MilliQ-Gradient system.

### 2.2. Preparation of Dietary Supplement Samples

The dietary supplements were encountered in the form of either capsules, tablets, or powders. For capsules, five items were weighed and opened and their contents were mixed and triturated in a mortar and pestle prior. Each dietary supplement purchased as powders, capsules, or tablets, about 1000 mg for powders and average weight in case of capsule content or tablets, were weighed into centrifuge tubes, re-suspended with methanol, and vortexed and sonicated for 30 min, following centrifugation for 15 min at 959× *g*. The procedure was repeated three times and the clear supernatant was subsequently transferred to a 10 mL volumetric flask. The final volume was adjusted with methanol to 10 mL and mixed thoroughly. Prior to injection, the samples were filtered through a 0.45 µm polytetrafluoroethylene (PTFE) membrane filter. Ultrasound-assisted extraction (UAE) was optimized using various solvents (methanol, ethanol, and water) and extraction durations (30 and 60 min). Methanol consistently yielded the highest extraction efficiency as it extracted both polar and non-polar compounds.

### 2.3. Liquid Chromatography-Quadrupole Time-of-Flight Mass Spectrometry (LC-QToF-MS)

LC-QToF-MS conditions and parameters were optimized to detect the maximum number of compounds.

The analytical methodology is the same as reported elsewhere [14,38,39,40]. The liquid chromatographic system is an Agilent Series 1290 and the mass spectrometric analysis was performed with a QToF-MS/MS (Model #G6530A, Agilent Technologies, Palo Alto, CA, USA) equipped with an ESI source with Jet Stream technology. All the operations, acquisition, and analysis of data were controlled by Agilent MassHunter Acquisition Software Ver. A.10.00 and operated under MassHunter Workstation software Ver. B.10.00. Each sample was analyzed in both positive and negative modes to provide abundant information for structural identification. Mass spectra are recorded across the range *m*/*z* = 50–1700 with accurate mass measurement of all mass peaks. MassHunter Workstation software, including Qualitative Analysis (version B.10.00), was used for processing both raw MS and MS-MS data, including molecular feature extraction, background subtraction, data filtering, and molecular formula estimation. The raw data were processed using the Find by Molecular Feature (MF) algorithm called Molecular Feature Extractor (MFE) within MassHunter Qualitative Analysis software. Extracted molecular features were processed to create a list of compounds.

A compound search for the non-targeted compounds was characterized by matching the experimental molecular formula in (a) The Agilent MassHunter Forensics and Toxicology (>9000 components) Personal Compound Database (PCD) and (b) In-House-generated library for 15,000 components of medicinal plant samples. Other search engines including SciFinder (web-based version), Dictionary of Natural product (CRC, 2023), and Google search engines by molecular formulae were used for the identification of “known unknowns”. These approaches have been utilized to identify a wide range of components, including additives, compounds from natural products, etc. The in-house library includes the compound name, molecular formula, exact mass, CAS #, and structure of each compound. From the possible positive hits, the results were compared with MS-MS experiments and with those available in the literature. All compounds either generated a high-abundance [M−H]^−^ or/and [M+HCOO]^−^ ion in negative mode or a high-abundance [M+H]^+^ or/and [M+Na]^+^ ion in positive mode; therefore, the [M−H]^−^ or [M+H]^+^ ions of each compound were selected as the precursor ions for subsequent MS-MS experiments to give more fragment ions. The generation of diagnostic fragment ions provides information concerning the core skeleton and nature of the substituents and helps in the identification of the compounds.

## 3. Results

### 3.1. Product Analysis

In total, 36 of the 44 products (82%) had inaccurate labels. Twenty-seven (61%) had ingredients listed on the Supplement Facts label not detected through analysis (i.e., misbranded). Missing ingredients ranged from 6% to 50% of all ingredients listed on any one product label. Twenty-two products listed either Yohimbe bark extract or yohimbine on the label. Of the 22, three were mislabeled (two listed yohimbine and analysis showed the presence of Yohimbe bark; one listed Yohimbe bark extract but analysis detected yohimbine alone). Sixteen products (36%) contained hidden ingredients not presented on the labels, such as berberine derivatives, choline, flavones, *Oryza sativa*, synephrine, theobromine, and tryptophan. While none of the products purchased on bases contained DoD Prohibited Ingredients, four products (representing 9% of the total products) purchased within one mile from a base listed multiple prohibited ingredients on the labels, and these ingredients were all detected, including DMHA, *Ephedra viridis*, higenamine, hordenine, octopamine, *N*-isopropylnorsynephrine, and sulbutiamine. Additionally, halostachine, 2-phenylethan-1-amine, or β-Phenylethylamine, were found on labels and detected in two of these products within one mile from a base, and one additional product on a base. These ingredients are prohibited in sport according to WADA; however, they are not currently DoD prohibited (Table 1).

### 3.2. Product Descriptions

A total of 40 out of 44 (91%) products scored a “no-go”, according to the OPSS Scorecard, an educational tool used to screen a supplement for safety and help mitigate risk based solely on what is presented on a label. None of the products had a BSCG Certified Drug-Free, Informed Sport, NSF Certified Sport, or USP-verified third-party certification seal present on the labels. Two products had seals that indicated them having been “quality tested” or having an “ironclad quality and guarantee”, but no such third-party organization was associated with such qualifications.

Twenty-eight (64%) products contained proprietary blends, matrices, or complexes, where the amount of each ingredient contained in a product was not disclosed on the product label. Thirty-nine (89%) products listed caffeine in combination with multiple other stimulants, such as evodiamine, green tea, guarana, rauwolscine, yerba mate, Yohimbe, and yohimbine. Twenty-one (48%) products listed the caffeine level at greater than 200 mg per serving (*n* = 17), with some up to 350 mg per serving; others did not disclose the amount contained in a proprietary blend (*n* = 4), all of which were combined with multiple other stimulants. The majority (*n* = 40 (91%)) had questionable claims or statements on the labels, such as “6X more weight loss”, “super strength technology”, “pharmaceutical grade fat burner”, and “advanced scientifically tested key ingredients”. Many products had claims not only for weight loss, but also for enhanced energy, mental focus, and concentration. The approximate cost for a 30-day supply of these products ranged from USD 11.98 to USD 89.98, with an average cost of USD 42.60.

**Figure 1:**
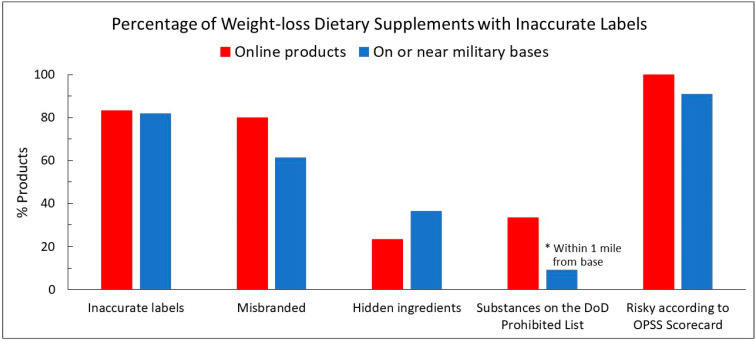
Results of analysis of weight-loss dietary supplements purchased online (*n* = 30) and on or near bases (*n* = 44). * These products were only those purchased within one mile from base.

Figure 1 depicts a comparison of the select weight-loss dietary supplements purchased for analysis from online websites offering military discounts to service members (*n* = 30) [14] with the current analysis of select weight-loss dietary supplements purchased on or near military bases across the country (*n* = 44). This offers a slight snapshot of the weight-loss dietary supplement landscape to which service members are exposed when looking for solutions to help manage their weight.

## 4. Discussion

The majority of the select weight-loss supplements sold on or near bases across the country had inaccurate labels, similar to our findings of those sold online—82% (36/44) vs. 83% (25/30), respectively. While there were more hidden ingredients detected in the current analysis from those found online, those online consisted of substances more likely to be DoD prohibited. Products within one mile from the base had substances listed on the labels that were detected through analysis and prohibited for military use. The majority of products scored as a “no-go” according to the OPSS Scorecard, similar to those found online. Service members need to be certain that a product is of a high quality, safe to consume, and free from prohibited substances and other performance-enhancing ingredients not allowed for use before making any product purchase. Beyond the potential harm to a service member’s health and career from consuming dangerous or adulterated products, the deceptive marketing of questionable claims not backed by science, advertising ingredients on a label but not contained in a product, or placing fictitious seals to imply quality all may impact the service member’s financial and mental fitness. Service members should not invest in such products and hope for a ‘magic bullet’ yet receive little in return. As the market soars, further action is needed to protect service members.

In recent years, many independent researchers have tested dietary supplement products readily available online or in retail stores [14,39,40,41,42,43,44,45]. The results show that the mislabeling and contamination of dietary supplements are ever-present, and weight-loss and other combination-type products have been found to contain hidden drugs and other substances not allowed in dietary supplements. A recent review collating results across such studies showed that of the 3132 analyzed dietary supplements, more than 28% pose a potential risk of unintentional doping from undisclosed substances banned by WADA [46]. The only way to know whether a dietary supplement label is accurate is to make sure the product has a seal present on the label from a reputable third-party organization. The marketing of adulterated or low-quality supplements would not make it to the marketplace if this were a requirement for all of the industry. 

Currently, no federal rules or regulations require third-party certification. However, some organizations, such as Major League Baseball (MLB), require their athletes to only use third-party-certified products. Unfortunately, many consumers are purchasing supplements online, and when asked, less than 40% consistently purchase those with third-party seals [47,48]. Most recently, Amazon announced that selling partners must verify supplement products via third-party testing to ensure they contain the ingredients claimed on the label and do not contain undeclared active pharmaceutical ingredients [49]. This is one step in the right direction for the industry to gain the trust that consumers need to ensure safe supplement use. However, what is considered third-party testing and the requirements that must be met need careful attention. While several reputable third-party companies offer testing and quality assurance, each has varying testing requirements. Moreover, some dietary supplement products have seals on their label that suggest that they have been quality tested by a third party when they have not. This is a problem.

In 2019, the Consortium for Health and Military Performance (a DoD program hosted at the Uniformed Services University), in collaboration with the US Anti-Doping Agency (USADA), Ultimate Fighting Championship (UFC), MLB, and the MLB Players Association, published a consensus statement on essential features of third-party certification programs for dietary supplements consumed by athletes and service members; the authors outlined ways in which third-party programs can further develop to improve access to safe, high-quality dietary supplements [50]. Highlighted in the consensus statement are third-party certification criteria, such as “The certification program should be accredited to ISO 17065”, “The certifier must be demonstrably impartial, conflict-free and competent to carry out the certification program”, and “The program certifies dietary supplements against NSF/ANSI 173-Dietary supplements standard”.

According to NSF/ANSI 173-2024 [51] (the current consensus standard in the US for analyzing and testing dietary supplements), caffeine at a level greater than 100 mg per serving should contain a warning on the dietary supplement label. In addition, it states “Supplements containing caffeine shall have caffeine content tested and verified. The amount consumed shall not exceed 200 mg per serving every 4 h and 800 mg/d”. According to this criterion alone, about half of the products analyzed in the current study could not have even undergone third-party testing due to their labels indicating more than 200 mg per serving of caffeine.

In addition, the ANSI 173 states “Products and ingredients deemed a hazard to public health or safety by a regulatory agency having jurisdiction shall be excluded from the scope of this document” [51]. Half of the products analyzed listed either yohimbe or yohimbine on the product labels. As with the current analysis, other studies have found these ingredients to be mislabeled and with the actual contents in excess of what is indicated on the labels [45,52]. Some countries have already banned these ingredients from dietary supplements due to safety concerns [52]. Yohimbine alone and when combined with other stimulants, such as caffeine, can lead to an increased heart rate and pose a cardiovascular risk, cause brain hemorrhage, and even resulting in death [20,53,54,55]. The select products we analyzed had multiple other stimulants beyond yohimbe and yohimbine combined with caffeine above 200 mg. So, the question begs, if dietary supplements are meant to supplement the diet, should such stimulants, or combinations of multiple stimulants, even be considered for inclusion in dietary supplements? 

### Limitations

This study only analyzed 40 products sold on 12 bases across the country and four products sold one mile from a base, so we do not know whether the label inaccuracies reflect the majority of products sold on or near the other 740 or so military bases [56]. We cannot generalize these results to the broader US or what other countries’ dietary landscape might look like. In addition, the amounts of each ingredient detected were not quantified as part of this study. Thus, we do not know whether the select products might indicate other concerns, such as a misrepresentation of the amounts of each ingredient contained in a product. Further, the analytical method has limitations when analyzing highly lipophilic (fat-soluble) compounds and minerals, due to the inefficient ionization of these molecules in the mass spectrometer, which leads to poor sensitivity and the inconsistent detection of such ingredients. Therefore, some ingredients presented on product labels were neither counted nor included in the analysis, as noted in Table 1.

## 5. Conclusions

This case series study analyzed 44 select weight-loss dietary supplements sold on or near US military bases across the country. The majority had inaccurate labels, some being misbranded with ingredients present on the labels but not detected through analysis, and some containing hidden ingredients not present on the labels. The majority scored a “no-go” according to the OPSS Scorecard. The label inaccuracy of products sold online as compared to those found on or near bases appears similar.

Third-party certification requirements for dietary supplements, specific to the DoD and leveraging standards already in place, will help protect service members who search for ways to improve their health and optimize their performance. DoD stakeholders and military establishments are working together with OPSS to establish effective ways for service members to have more third-party-certified products available on military bases. Continued education within and outside the DoD is critical to ensure safe dietary supplement use.

## Figures and Tables

**Table 1:** Product analysis of 44 weight-loss dietary supplements purchased on or near military bases.

Product No.	# of Ingredients Presented on Product Label	Ingredients on Label Analyzable but Not Detected, No (%) ^a^	Additional or Hidden Components Detected Not Present on Label	DoD Prohibited Ingredients ^b^ or WADA S6 Stimulants ^c^ Detected
Product label verification: Accurate				
5	6	None	None	None
10	10	None	None	None
21	12	None	None	None
22	6	None	None	None
26	12	None	None	None
29 ^d^	9	None	None	DMHA ^b^
34	13	None	None	None
40	6	None	None	None
Product label verification: Not accurate				
1	14	2 (20)	None	None
2	6	None	Synephrine	None
3	16	2 (14)	None	None
4	57	6 (19)	None	None
6	11	5 (46)	None	None
7	8	2 (33)	None	None
8	18	1 (6)	None	None
9	7	None	*O*-polymethoxylated flavones including tangeretin, poncirin, and senesetin	None
11	8	2 (25)	None	None
12	8	2 (25)	None	None
13	27	4 (15)	None	None
14	6	None	Synephrine	None
15	14	2 (20)	None	None
16	9	None	Theobromine	None
17	14	1 (8)	Choline	None
18	12	None	Tryptophan	None
19	14	4 (36)	None	None
20	25	3 (13)	None	None
23	6	1 (17)	None	None
24	9	1 (13)	None	None
25	14	1 (8)	None	None
27	28	5 (19)	None	None
28	12	1 (13)	Pyridoxic acid lactone; Tryptophan	None
30 ^d^	12	4 (36)	Synephrine	Ephedra (*Ephedra viridis*) ^b^ 2-phenylethan-1-amine ^c^
31 ^d^	29	9 (32)	None	DMHA ^b^ Higenamine ^b^ Hordenine ^b^ Sulbutiamine ^b^ Octopamine ^b^ *N*-isopropylnorsynephrine ^b^ β-Phenylethylamine ^c^ Halostachine ^c^
32 ^d^	5	1 (20)	None	DMHA ^b^ Hordenine ^b^
33	11	None	Tyrosine derivative; Homocitric acid; Hydroxy maltol; Maltol	None
35	9	1 (13)	None	None
36	12	None	Tryptophan	None
37	14	1 (8)	Choline	None
38	16	1 (7)	None	None
39	5	None	Polyethylene glycols (PEGs)	None
41	12	6 (50) ^e^	*Oryza sativa*	None
42	11	1 (10)	Berberine derivatives including berberine, methylberberine, oxoberberine	None
43	6	2 (33)	Dihydrocapsicin	None
44	12	None	Polymethoxylated flavones including senesetin, tangeretin, nobiletin	Halostachine ^c^

^a^ The number of analyzable ingredients differs from the number of ingredients presented on the product label due to the instrumentation limits for analysis. Elements including zinc, selenium, calcium, chromium, manganese, magnesium, potassium, sodium, and so forth are not able to be analyzed, as well as fat-soluble vitamins, including Vitamin D3. ^b^ DoD Prohibited Dietary Supplement Ingredients List. ^c^ WADA S6 stimulants: Prohibited in competition. ^d^ Stores within one mile from base. ^e^ Trace elements found in 3 of 6.

## Data Availability

The original contributions presented in this study are included in the article. Further inquiries can be directed to the corresponding author. The data are not publicly available due to legal reasons.
